# Divergent Strategies in Innate Immune Evasion: A Comparative Review of Three Alphaherpesvirus Subfamily Members—PRV, HSV‐1, and VZV

**DOI:** 10.1155/tbed/6236477

**Published:** 2026-10-08

**Authors:** Enping Liu, Jiayi Xue, Mengting He, Yumei Chen, Chao Liang, Jingming Zhou, Aiping Wang

**Affiliations:** ^1^ The School of Life Sciences Zhengzhou University Zhengzhou 450001 China zzu.edu.cn; ^2^ Longhu Laboratory Zhengzhou 450002 China; ^3^ Henan Key Laboratory of Immunobiology Zhengzhou 450001 China

**Keywords:** HSV, immune evasion, innate immune, PRV, VZV

## Abstract

Pseudorabies virus (PRV), an important member of the Alphaherpesvirinae subfamily, shares high evolutionary homology with other members of the same subfamily, including human herpes simplex virus (HSV) and varicella‐zoster virus (VZV). However, significant differences exist among them in terms of host range, pathogenicity, and immune evasion strategies. This review systematically compares the roles of PRV, HSV type 1 (HSV‐1), and VZV in modulating host innate immune responses, encompassing the cyclic GMP‐AMP synthase (cGAS)‐stimulator of interferon genes (STING) signaling, RIG‐I‐like receptor (RLR) signaling, NOD‐like receptor (NLR) signaling, Toll‐like receptor (TLR) signaling, Janus kinase (JAK)‐signal transducer and activator of transcription (STAT) pathway, apoptosis, and autophagy. By summarizing and comparing the interactions of these three viruses with the host innate immune response, this review aims to provide a theoretical reference for understanding the conserved and unique mechanisms of immune evasion among alphaherpesviruses, and to offer insights for the development of novel antiviral strategies.

## 1. Introduction

The subfamily Alphaherpesvirinae is a subfamily of the family Herpesviridae, comprising five genera and ~45 virus species. Taxonomically, herpes simplex virus type 1 (HSV‐1) and HSV type 2 (HSV‐2) are classified into the genus Simplexvirus, whereas pseudorabies virus (PRV) and varicella‐zoster virus (VZV) belong to the genus Varicellovirus. Further sequence comparison studies have shown that PRV and HSV‐1 are placed on distal branches of the alphaherpesvirus phylogenetic tree, with their genomes sharing only ~8% sequence homology. In contrast, PRV and VZV, both belonging to the same genus Varicellovirus, share a more recent common ancestor and are more closely related to each other than either is to HSV [1]. Nevertheless, the genes of these three viruses share many similarities in the genomic arrangement and function. These viruses have a short replication cycle, can infect humans and other mammals, establish lifelong latency in sensory neurons after acute infection, and possess the ability to reactivate periodically, posing a significant threat to public health [2, 3].

PRV, also known as suid alphaherpesvirus type 1 (SuHV‐1), is a member of the Alphaherpesvirinae subfamily and the causative agent of Aujeszky’s disease. The disease primarily infects swine, causing substantial economic losses to the global pig industry. Notably, PRV has an exceptionally broad host range among alphaherpesviruses, capable of infecting various mammals, including cattle, sheep, dogs, cats, and rodents, leading to fatal neurological diseases [4]. Humans are considered accidental hosts, with rare but severe cases of encephalitis and endophthalmitis reported in recent years [5]. PRV shares extensive genetic and structural homology with other members of the Alphaherpesvirinae, especially human HSV and VZV. Its viral genome is ~154 kb of double‐stranded DNA, encoding more than 70 viral proteins [6]. HSV‐1 primarily causes oral HSV disease, infectious corneal blindness, fatal sporadic encephalitis, and genital herpes in humans. Its genome is ~152 kb in length, encoding over 80 viral proteins [7]. VZV primarily causes chickenpox in children, establishes latency in ganglia, and can be reactivated in the elderly or immunocompromised individuals, causing shingles. Its genome is ~125 kb, encoding more than 71 viral proteins [8]. It has been established that the sequences and functions of proteins encoded by these three viruses are conserved to some extent, particularly in processes such as viral replication, latency establishment, and immune evasion (Table S1).

In recent years, significant progress has been made in elucidating the molecular mechanisms by which alphaherpesviruses evade host innate immunity. Conserved viral proteins within this subfamily, such as US3 [9], UL21 [10], and ICP0 (EP0) [11], have been increasingly discovered to target key nodes of the innate immune signaling network and modulate functions across multiple innate immune pathways (including host innate immune signaling, apoptosis, and autophagy) through various mechanisms. Before systematically comparing the immune evasion mechanisms of the three viruses, it is necessary to first acknowledge an objective reality: although PRV, HSV‐1, and VZV all belong to the Alphaherpesvirinae subfamily, there is a significant asymmetry in the depth of research on them. As a virus that is broadly host‐ranged, has low human pathogenicity, and shares high homology with HSV and VZV, PRV is often used as a classic model system for molecular virology studies of alphaherpesviruses. A complete research chain has been established, ranging from in vitro cell models to natural host (swine) infection models, and its immune evasion mechanisms have been characterized down to the single‐amino‐acid level. HSV‐1 has received extensive attention due to its direct association with human diseases, particularly in the areas of latent infection and reactivation, where substantial data have been accumulated. In contrast, research on VZV has been constrained by its strict human tropism and the lack of suitable small‐animal models; to date, only about 10% of its ORFs have been found to function in immune regulation. Since this review uses PRV, a model virus, as its entry point, PRV‐related research occupies a relatively large proportion of the text; however, this does not mean that research on PRV is the most thorough and extensive. To keep the comparative analysis fair while focusing on systematically presenting the comprehensive mechanisms of PRV, this article also systematically compiles representative findings on HSV‐1 and VZV, and in Sections 6and 7, centrally discusses the key unresolved questions common to all three viruses, aiming to enrich the content as much as possible and provide references for future research.

This review aims to systematically compare the similarities and differences in innate immune evasion strategies among PRV, HSV‐1, and VZV based on existing research findings. By analyzing the conserved and divergent mechanisms of immune regulation among these viruses, this review seeks to provide a theoretical foundation for a deeper understanding of alphaherpesvirus–host interactions and to offer insights for the development of novel antiviral intervention strategies.

## 2. Evasion of Antiviral Responses Mediated by Diverse Innate Immune Receptors by PRV, HSV‐1, and VZV

The host innate immune system detects viral infections and initiates antiviral responses through a diverse array of pattern recognition receptors (PRRs), including Toll‐like receptors (TLRs), RIG‐I‐like receptors (RLRs), NOD‐like receptors (NLRs), and cytosolic DNA sensors [12]. As DNA viruses, alphaherpesviruses such as PRV, HSV‐1, and VZV generate multiple molecular patterns during their replication cycle that are potentially recognizable by these PRR families. To establish successful infection, these viruses have evolved sophisticated strategies to evade or disrupt PRR‐mediated antiviral signaling. This chapter will summarize and compare how PRV and other alphaherpesviruses antagonize antiviral responses mediated by distinct innate immune receptors, thereby evading host defenses and achieving successful invasion and replication.

### 2.1. Cyclic GMP‐AMP Synthase (cGAS)‐Stimulator of Interferon Genes (STING) Signaling Pathway

cGAS is the primary cytosolic DNA sensor. Upon recognizing double‐stranded DNA, cGAS is activated and synthesizes the second messenger 2^′^3^′^‐cyclic GMP‐AMP (2^′^3^′^‐cGAMP). This messenger binds and activates the STING. Upon activation, STING translocates from the endoplasmic reticulum to the Golgi apparatus, where it binds and activates TANK‐binding kinase 1 (TBK1) and IκB kinase (IKK), which subsequently phosphorylate interferon regulatory factor 3 (IRF3) and nuclear factor of kappa light polypeptide gene enhancer in B‐cells inhibitor, alpha (IκBα), respectively. The phosphorylated IRF3 and nuclear factor kappa‐light‐chain‐enhancer of activated B cells (NF‐κB) dimers then enter the nucleus to initiate the transcription of type I interferons (IFNs) and inflammatory cytokines (TNF, IL6, IL‐1β, etc.), serving as a host antiviral defense mechanism [13]. Alphaherpesviruses are DNA viruses that can be recognized by cGAS in the cytoplasm upon host infection. Within the alphaherpesvirus family, PRV, HSV‐1, and VZV have all evolved multiple strategies targeting different nodes of the cGAS‐STING signaling pathway to achieve immune evasion, yet the depth and breadth of mechanistic studies vary significantly among the three viruses.

PRV employs multiple viral proteins to finely regulate this pathway at multiple levels: in targeting cGAS, UL42 binds to the DNA‐binding domain of cGAS, inhibiting its recognition of dsDNA and dimerization activation [14]; US1 from PRV, HSV‐1, and VZV directly interacts with cGAS and suppresses its dsDNA‐binding and enzymatic activity, with conserved overlapping peptides identified in PRV and HSV‐1 US1 proteins [15]; UL21 recruits the E3 ligase ubiquitin protein ligase E3C (UBE3C) to mediate K27‐linked ubiquitination of cGAS, leading to its recognition and degradation by the selective autophagy receptor Toll‐interacting protein (TOLLIP), a mechanism conserved in HSV‐1 [10]; UL49 (VP22) inhibits cGAS activity by preventing the degradation of the host RNA helicase DDX21, thereby enhancing the interaction between DDX21 and cGAS [16]; HSV‐1 UL49 also inhibits cGAS function through binding, although the precise mechanism remains unclear [17]. Additionally, PRV ICP0, despite being widely referred to as a homolog of HSV‐1 ICP0, is actually transcribed from a different genomic locus (EP0) and has been shown to facilitate cGAS degradation via the AKT‐mTOR‐dependent autophagy‐lysosomal pathway [18]. In targeting STING, PRV UL13 serves as an adaptor to hijack Ring finger protein 5 (RNF5), which conjugates noncanonical K27/K29‐linked ubiquitin chains onto STING, targeting it for proteasomal degradation rather than lysosomal clearance, thereby evading host innate immunity [19]; UL38 recruits TOLLIP to degrade STING [20]; US2 recruits Tripartite motif‐containing protein 21 (TRIM21) to mediate K48‐linked ubiquitination of STING for proteasomal degradation [21]; UL48 recruits TRIM21 to catalyze K33/K63‐linked ubiquitination of STING, leading to its recognition by Calcium‐binding and coiled‐coil domain‐containing protein 2/Nuclear dot protein 52 (CALCOCO2/NDP52) and subsequent lysosomal degradation, a function conserved in HSV‐1 and Cercopithecine herpesvirus 2 (CHV‐2) [22]; ICP0 also degrades STING via the autophagy‐lysosome pathway [18]. In targeting downstream transcription factors, UL13 phosphorylates IRF3 to inhibit IFN‐β production [19]; UL24 degrades IRF7 via the proteasome pathway [23]; and virion host shutoff (VHS) protein (encoded by UL41) recruits TOLLIP to degrade IRF3 [24]. Moreover, UL41 (VHS) is increasingly recognized for its pleiotropic functions, which enable it to target distinct substrates through pathway‐specific mechanisms.

In comparison, studies on HSV‐1 immune evasion mechanisms are more extensive and involve more novel mechanisms: in addition to mechanisms conserved with PRV such as UL21 and US1, HSV‐1 also encodes UL56, which inhibits cGAS binding to dsDNA and its enzymatic activity—a function conserved in VZV, human cytomegalovirus (HCMV), and others—and also suppresses the expression of cGAMP channel proteins [25]; UL37 attenuates cGAMP synthesis by deamidating cGAS [26]; ICP8 suppresses antiviral IFN production by reducing cGAMP levels in response to dsDNA stimulation [27]; UL41 (VHS) utilizes its intrinsic RNase activity to degrade cGAS [28]; STING plays a pivotal role during HSV‐1 infection, and the maintenance of STING stability requires the viral proteins immediate‐early protein ICP0 and US3 [29]. UL36 removes K63‐linked ubiquitin chains from STING via its deubiquitinase activity, inhibiting downstream IRF3 and IFN activation [30]; UL26 encodes the N‐terminal protease domain VP24, which blocks IRF3 binding to TBK1 [31]; ICP27 (a function also shared by its HSV‐2 homolog, though the PRV homolog UL54 does not exhibit this activity) inhibits IFN‐β production by interacting with the STING‐TBK1 complex [32]; and UL24 blocks NF‐κB subunit nuclear translocation [33].

Research on VZV remains the most limited, in addition to the functionally homologous proteins mentioned above, with only ORF9 identified to inhibit cGAMP production through interaction with cGAS [34], and it has been demonstrated that VZV gE suppresses the translocation of STING, consequently impairing the STING‐mediated IFN response [35].

Overall, PRV exhibits systematic, multinode, multilevel regulation of the cGAS‐STING pathway, HSV‐1 exhibits remarkable diversity in its immune evasion mechanisms, with the largest number of viral proteins identified to date, while VZV research is still in its early stages (Figure 1).

**Figure 1:**
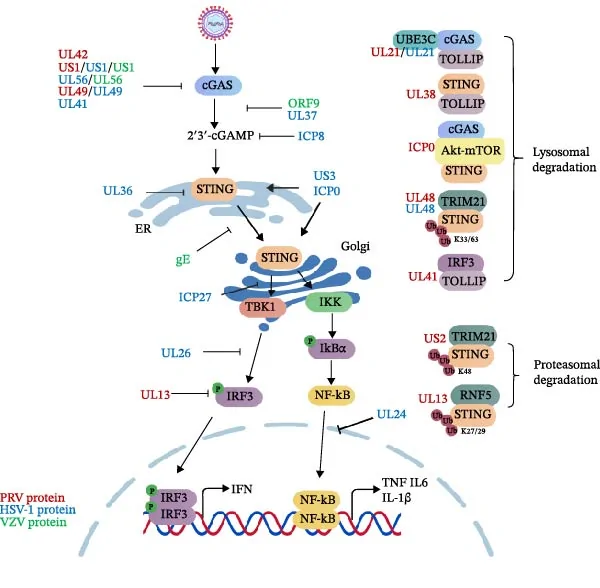
Schematic overview of the cGAS‐STING signaling pathway and its inhibition by PRV, HSV‐1, and VZV. Viral dsDNA in the cytosol is sensed by cGAS, which catalyzes the production of 2^′^3^′^‐cGAMP. This second messenger activates STING at the ER, triggering its translocation via the ER‐Golgi intermediate compartment (ERGIC) to the Golgi, where STING recruits and phosphorylates TBK1. The TBK1‐STING complex subsequently phosphorylates IRF3, promoting its dimerization and nuclear translocation, while simultaneously activating the IKK/NF‐κB axis. These events cooperatively induce type I IFNs and pro‐inflammatory cytokines. PRV: UL42 binds the DNA‐binding domain of cGAS to block dsDNA recognition and dimerization; US1 from PRV, HSV‐1, and VZV directly interacts with cGAS and suppresses its dsDNA‐binding and enzymatic activity, with conserved overlapping peptides identified in PRV and HSV‐1 US1 proteins. UL21 recruits UBE3C to mediate K27‐linked ubiquitination of cGAS, leading to TOLLIP‐mediated selective autophagic degradation (mechanism conserved in HSV‐1); UL49 (VP22) inhibits cGAS activity by preventing DDX21 degradation and enhancing DDX21–cGAS interaction (functionally similar to HSV‐1 UL49); ICP0 promotes degradation of cGAS and STING via the AKT‐mTOR‐dependent autophagolysosomal pathway. UL13 recruits RNF5 to mediate K27‐ and K29‐linked ubiquitination of STING and proteasomal degradation; UL38 recruits TOLLIP to degrade STING; US2 recruits TRIM21 to mediate K48‐linked ubiquitination and proteasomal degradation of STING; UL48 recruits TRIM21 to catalyze K33‐ and K63‐linked ubiquitination of STING, leading to lysosomal degradation (function conserved in HSV‐1); UL13 phosphorylates IRF3 to suppress IFN‐β production; UL41 (VHS) recruits TOLLIP to degrade IRF3. HSV‐1: In addition to the shared mechanisms with PRV, HSV‐1 UL56 inhibits cGAS binding to dsDNA and its enzymatic activity (function conserved in VZV and HCMV), and also suppresses cGAMP channel protein expression to block signaling; UL41 degrades cGAS via its RNase activity; UL37 deamidates cGAS to attenuate cGAMP synthesis; ICP8 reduces cGAMP levels; ICP0 and US3 are critical for maintaining STING stability during HSV‐1 infection; UL36 removes K63‐linked ubiquitin chains from STING via its deubiquitinase activity to inhibit downstream signaling; UL26 blocks the interaction between IRF3 and TBK1; ICP27 inhibits STING‐TBK1 complex formation; UL24 blocks nuclear translocation of NF‐κB subunits. VZV: ORF9 binds cGAS to inhibit cGAMP production; gE inhibits STING translocation.

### 2.2. TLR Signaling Pathway

TLRs are important PRRs in the host innate immune system. Based on their subcellular localization, TLRs can be divided into two categories: one group is expressed on the cell surface, including TLR2, TLR4, TLR5, and TLR6, which primarily recognize bacterial and viral envelope proteins; the other group is expressed intracellularly, including TLR3, TLR7, TLR8, and TLR9, which mainly recognize pathogen nucleic acids, with TLR9 being capable of recognizing viral DNA [36]. Upon recognition of viral nucleic acids, TLRs recruit the adaptor proteins myeloid differentiation primary response 88 (MyD88) or TIR‐domain‐containing adapter‐inducing IFN‐β (TRIF), subsequently activating signaling pathways such as NF‐κB, IRF3, and IRF7, leading to the production of antiviral substances including IFNs and inflammatory cytokines [37]. The TLR signaling pathway plays an important defensive role against alphaherpesvirus infections, and in turn, viruses have evolved various immune evasion strategies to interfere with this pathway.

Studies on PRV have shown that the TLR2/MyD88 axis is essential for PRV‐induced NF‐κB activation and the expression of downstream pro‐inflammatory cytokines. Meanwhile, the mitogen‐activated protein kinase (MAPK)/c‐Jun N‐terminal kinase (JNK) and phosphoinositide 3‐kinase (PI3K)/protein kinase B (Akt) signaling pathways are also activated through the TLR2/MyD88 axis and participate in regulating the pro‐inflammatory cytokine expression induced by PRV infection [38]. Furthermore, studies have confirmed that PRV infection can trigger the activation of the TLR4/NF‐κB signaling pathway, thereby mediating inflammatory responses [39]. Further research has revealed that PRV induces the secretion of bioactive inflammatory factors by activating three signaling pathways—the TLR‐NF‐κB axis, the absent in melanoma 2 (AIM2) inflammasome, and gasdermin D (GSDMD)—thereby inhibiting viral replication, a mechanism that plays an important role in the host defense against PRV infection [40]. Using a dual model of trigeminal ganglion primary cell infection and mouse intranasal infection, the researchers also found that PRV infection alters the gene and protein expression levels of immune signaling molecules such as TLR3, TRIF, TBK1, and IRF3, while suppressing IκBα protein expression during the late stage of infection. These results indicate that PRV can both activate antiviral immune responses through the TLR3‐TRIF‐TBK1‐IRF3 signaling axis and suppress IFN‐α expression in trigeminal ganglion cells via the same pathway, demonstrating a bidirectional immune regulation strategy of “both activation and inhibition.” However, the specific viral protein mediating this bidirectional regulatory effect and its direct molecular interaction with TLR3 pathway components have not yet been identified [41]. In summary, no PRV‐encoded specific viral protein directly targeting the TLR signaling pathway has been discovered to date.

For HSV, recent studies have revealed that HSV‐1 infection of microglial cells can downregulate the Golgi matrix protein 130 (GM130), disrupting Golgi structural integrity and thereby inhibiting TLR3‐mediated antiviral immune responses [42]. Additionally, the HSV‐1 immediate‐early protein ICP0 can reduce TLR2‐mediated inflammatory responses during HSV‐1 infection, a function that depends on its E3 ubiquitin ligase activity [43]. This study suggests that ICP0 may inhibit TLR2 signaling by degrading MyD88, Mal, and other adaptor or signaling molecules, but the specific mechanism requires further investigation. Furthermore, US3 also suppresses TLR2‐mediated NF‐κB signaling, although its site of action is only defined as being between MyD88 and p65, and the specific target remains unclear [44].

Research on the regulation of host TLR immune signaling by VZV has primarily focused on the TLR2 and TLR3 signaling pathways. The study by Wang et al. [45] is the first to confirm that VZV can activate inflammatory cytokine responses through TLR2. The research by Black et al. [46] revealed the impact of VZV infection on the TLR3 signaling pathway in keratinocytes, demonstrating that VZV interferes with multiple signaling pathways, including TLR3, to inhibit the antigen‐presenting function of keratinocytes, thereby evading adaptive immune responses. In 2022, Doratt et al. [47] showed that in lung epithelial cells, multiple antiviral sensor molecules and IFN signaling pathway components, including TLR3, participate in restricting VZV replication. Of note, no VZV‐encoded specific viral protein directly targeting the TLR signaling pathway has been identified to date. Existing evidence primarily focuses on the effects of VZV infection on TLR signaling pathways, while the specific molecular mechanisms of viral immune evasion remain to be further investigated.

In summary, mechanistic studies on the regulation of the TLR pathway by all three viruses remain relatively superficial. Although research on PRV in regulating this pathway is comparatively extensive, exhibiting a bidirectional regulatory strategy that both activates inflammatory responses and suppresses type I IFN production, the findings have largely been confined to phenotypic and pathway‐level descriptions—such as identifying which molecules are activated—without elucidating which viral protein is responsible for the regulation or through what molecular mechanism (e.g., direct binding, degradation, or posttranslational modification) this regulation is achieved.

### 2.3. RLR Signaling Pathway

The RLR family consists of three members: RIG‐I, melanoma differentiation‐associated gene 5 (MDA5), and laboratory of genetics and physiology 2 (LGP2), all of which are localized in the cytoplasm and contain a central helicase domain and a C‐terminal domain (CTD) essential for RNA recognition [48]. Among them, RIG‐I and MDA5 also possess a tandem caspase recruitment domain (CARD) at their N‐terminus, through which they bind to the CARD of the downstream adaptor protein mitochondrial antiviral signaling protein (MAVS), recruiting downstream signaling molecules and subsequently activating IRF3 and NF‐κB via the TNF receptor‐associated factor (TRAF) family and the IKK family members, leading to the induction of IFNs and pro‐inflammatory cytokines [49]. Although RLRs primarily recognize RNA viruses, recent studies have revealed that DNA viruses (e.g., PRV, HSV, and VZV) produce RNA intermediates during replication that can also activate RLRs [50–52]. In response, these viruses have evolved various regulatory strategies: in PRV, UL24 inhibits RIG‐I‐induced oligoadenylate synthetase‐like (OASL) expression in an IRF3‐dependent manner [53], while UL13 downregulates the transcription of RIG‐I and MDA5 by suppressing NF‐κB activation [51]. In HSV, the γ134.5 gene [54] product, ICP34.5, promotes HSV growth by blocking the assembly of RIG‐I with 14‐3‐3ε, thereby preventing RIG‐I mitochondrial translocation [55], US11 protein interacts with RIG‐I and MDA5 to suppress downstream signaling [56], UL31 protein inhibits the formation of the IKK‐related kinase i (IKKi)‐IRF3 complex [57], the N‐terminal deubiquitinase domain of UL36 protein inhibits IFN‐β promoter activity through deubiquitination of TRAF3 (with homologs present in PRV and other herpesviruses, though their functions remain to be elucidated) [58, 59], and US3 protein inhibits TRIM25‐mediated ubiquitination and RIG‐I‐MAVS binding by phosphorylating RIG‐I, and the phosphorylation function of the US3 protein on RIG‐I has been preliminarily validated in various alphaherpesviruses, including HSV‐2 and VZV [60]. In contrast, apart from US3, research on the regulation of host RLR signaling by VZV remains largely unexplored. Overall, as a group of DNA viruses, the regulation of RLR signaling by alphaherpesviruses is still in its early stages and awaits further exploration (Figure 2).

**Figure 2:**
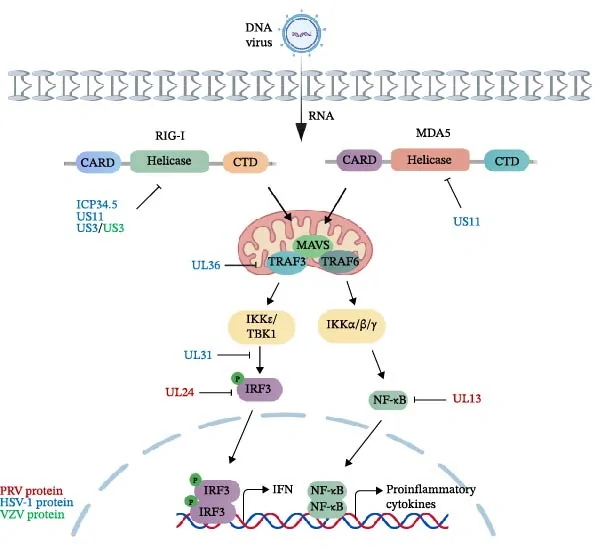
Schematic overview of the RLR signaling pathway and its inhibition by PRV, HSV‐1, and VZV. Cytosolic RIG‐I and MDA5 recognize viral RNA produced during viral replication via their CTD domains and subsequently activate the downstream adaptor MAVS through their CARD domains. MAVS recruits TRAF3/TRAF6 and IKK family members, which then activate IRF3 and NF‐κB to induce type I IFNs and pro‐inflammatory cytokines. In PRV, UL24 suppresses RIG‐I‐induced immune responses in an IRF3‐dependent manner, whereas UL13 downregulates RIG‐I/MDA5 transcription by inhibiting NF‐κB activation. In HSV‐1, the ICP34.5 promotes HSV‐1 growth by blocking the assembly of RIG‐I with 14‐3‐3ε, thereby preventing RIG‐I mitochondrial translocation. US11 interacts with RIG‐I and MDA5 to inhibit downstream signaling; UL31 inhibits the formation of the IKKi‐IRF3 complex; the N‐terminal deubiquitinase domain of UL36 suppresses IFN‐β promoter activity by deubiquitinating TRAF3; and US3 inhibits TRIM25‐mediated ubiquitination and RIG‐I‐MAVS binding by phosphorylating RIG‐I. Notably, the phosphorylation of RIG‐I by US3 has also been preliminarily validated in VZV.

### 2.4. NF‐κB Signaling Pathway

The NF‐κB family consists of five members—p50, p52, p65 (RelA), c‐Rel, and RelB—which form homo‐ or heterodimers through a conserved N‐terminal Rel homology domain [61]. In resting cells, these dimers are inhibited by IκBα protein; upon activation of the canonical pathway, the NF‐κB essential modulator (NEMO)‐containing IKK complex induces phosphorylation and degradation of IκBα, thereby releasing dimers such as p65:p50 [62]. Signaling pathways including cGAS‐STING, TLR, RLR, tumor necrosis factor receptor (TNF‐R), and interleukin‐1 receptor (IL‐1R) can all activate NF‐κB, among which signals from TLR, IL‐1R, and TNF‐R converge at the common IKK complex to phosphorylate IκBα [12]. Previous sections have described the regulation of NF‐κB signaling by cGAS‐STING, TLR, and RLR receptors; this section focuses on TNF‐R‐mediated NF‐κB signaling.

In PRV, ICP0 directly binds p65 and degrades it via the proteasome pathway, thereby blocking p65 phosphorylation and nuclear translocation [63]. In contrast, the HSV‐1 ICP0 protein utilizes its intrinsic E3 ubiquitin ligase activity to degrade p50 and prevent p65 from entering the nucleus [64], highlighting a functional divergence between these two related proteins. PRV UL24 inhibits this signaling by degrading p65, though the specific E3 ubiquitin ligase involved remains to be identified [65]. PRV UL41 (VHS) is an IFN‐I resistance factor with RNase activity that degrades p50 through autophagy and apoptosis [24]. Apart from the conserved proteins mentioned above, in HSV‐1, the US3 kinase phosphorylates p65 at serine 75 and blocks its nuclear import, thereby inhibiting TNF‐α‐induced NF‐κB activation [66]. UL42 interacts with both p50 and p65 to inhibit TNF‐α‐induced NF‐κB activity, while VP16 blocks NF‐κB signaling through interaction with p65 [67]. ICP27 inhibits TNF‐α‐induced NF‐κB activation via two mechanisms: on the one hand, it binds to IκBα, reducing its phosphorylation by various IKKs and thus stabilizing IκBα [68]; on the other hand, it enhances the interaction between Daxx (a protein that binds the Fas receptor death domain and inhibits NF‐κB transcription factors) and p65, thereby suppressing p65 acetylation [69]. UL2 attenuates p65 phosphorylation at Ser536 and inhibits TNF‐α‐induced NF‐κB activation [70]. HSV‐2 ICP22 directly interacts with p65 to inhibit its phosphorylation and nuclear transport, thereby blocking NF‐κB activation. This study also suggests that ICP22 proteins from HSV‐1, VZV, and PRV can inhibit TNF‐α‐induced NF‐κB activation, though the specific mechanisms remain unclear [71]. In VZV, ORF61 has been found to block TNF‐α‐induced NF‐κB activation by inhibiting the ubiquitination and degradation of IκBα [72]. Overall, compared with PRV and VZV, the regulatory mechanisms of HSV in this signaling pathway are more extensively studied.

Based on current research findings, the targets of PRV, HSV, and VZV in inhibiting TNF‐R‐mediated NF‐κB signaling are primarily focused on the NF‐κB subunits p50 and p65, as well as IκBα (Figure 3).

**Figure 3:**
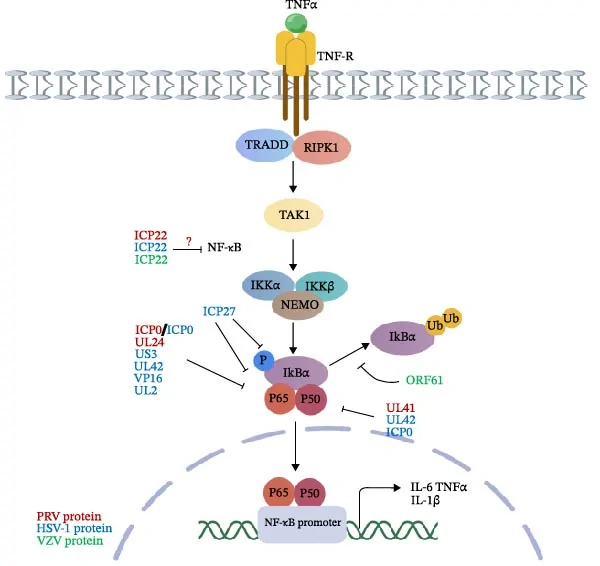
Schematic overview of the TNFα‐induced NF‐κB signaling pathway and its inhibition by PRV, HSV‐1, and VZV. TNFα activates TNFR on the plasma membrane, recruiting TRADD and RIPK1 to activate TAK1. Through the IKK complex and NEMO, IκBα is phosphorylated and degraded, releasing the NF‐κB dimer (p65/p50) for nuclear translocation and cytokine transcription. To counteract this host response, alphaherpesviruses deploy diverse viral proteins that target distinct steps of the pathway. PRV: ICP0 directly binds p65 and degrades it via the proteasome pathway, thereby blocking p65 phosphorylation and nuclear translocation. UL24 degrades p65 via the ubiquitin‐proteasome pathway; UL41 (VHS) degrades p50 through autophagy and apoptotic pathways via its RNase activity. HSV‐1: ICP0 degrades p50 via its E3 ubiquitin ligase activity and blocks p65 nuclear import; US3 phosphorylates p65 at Ser75 to block its nuclear import; UL42 binds both p50 and p65 to suppress NF‐κB activity; VP16 binds p65; ICP27 stabilizes IκBα by reducing IκBα phosphorylation and enhances Daxx–p65 interaction to inhibit p65 acetylation; UL2 attenuates p65 Ser536 phosphorylation. Conserved but mechanistically uncharacterized: the ICP22 homologs in HSV‐1, VZV, and PRV suppress NF‐κB function, though the underlying mechanisms remain unknown. VZV: ORF61 blocks TNFα‐induced NF‐κB activation by inhibiting IκBα ubiquitination and degradation.

## 3. IFN Alpha/Beta Receptor (IFNAR)–Janus Kinase (JAK)–Signal Transducer and Activator of Transcription (STAT) Signaling Pathway

Type I IFNs, such as IFN‐α/β, are recognized by the IFNAR complex IFNAR1‐IFNAR2. Subsequently, they activate antiviral signaling through the JAK‐STAT pathway [73]. The JAK family consists of four cytoplasmic tyrosine kinases (JAK1, JAK2, JAK3, and TYK2), which link the intracellular regions of IFN receptors. The STAT family comprises seven intracellular transcription factors: STAT1, STAT2, STAT3, STAT4, STAT5a, STAT5b, and STAT6. Upon binding of IFN‐α/β to IFNAR, the receptor dimerizes, activating the receptor‐associated JAK1 and tyrosine kinase 2 (TYK2). JAKs phosphorylate tyrosine residues in the intracellular domain of IFNAR, creating docking sites for STAT1/STAT2. STAT1/STAT2 are then phosphorylated by JAKs. The phosphorylated STAT1/STAT2 combine with IRF9 to form the IFN‐stimulated gene factor 3 (ISGF3) transcription factor complex. ISGF3 translocates into the nucleus, binds to the IFN‐stimulated response element (ISRE), and initiates the transcription of antiviral IFN‐stimulated genes (ISGs) [74]. Classical ISGs include ISG15, ISG20, myxovirus resistance (Mx) genes, IFN‐induced transmembrane protein (IFITM), 2^′^‐5^′^‐oligoadenylate synthetase (OAS), and protein kinase R (PKR) [75]. Since IFN stimulation of the antiviral response critically jeopardizes virus survival, viruses employ specific strategies such as proteasomal degradation and dephosphorylation, targeting the JAK/STAT pathway to counteract the host’s innate immune system.

In PRV, UL50 interferes with IFNα‐induced JAK‐STAT signaling. Specifically, UL50 degrades IFNAR2 via the proteasome pathway through its CTD, which is independent of dUTPase activity, thereby blocking antiviral signaling. This function has also been validated in HSV‐1 UL50, although its inhibitory activity is weaker compared to that of PRV UL50 [76]. Yin et al. [77] reported that PRV infection degrades JAK1 and TYK2 via the proteasome pathway, hindering signal transduction, marking the first discovery that an alphaherpesvirus inhibits the IFN signaling pathway by degrading these two JAKs. PRV VHS protein binds to the conserved sequences KUUUCY and CSDGGA in the mRNAs of TYK2, STAT1, and IRF9 and degrades these mRNAs through its ribonuclease activity, leading to reduced antiviral activity. These findings highlight the importance of the conserved amino acid residue F78 in UL41 (VHS), which may serve as a key functional residue in the VHS proteins of other herpesviruses [78]. UL42, a DNA polymerase processivity factor, directly interacts with the ISRE through its DNA‐binding domain, thereby blocking the binding of ISGF3 to the ISRE. This function of UL42 is conserved in PRV and HSV‐1, with the difference being the four DNA‐binding sites through which the protein exerts its function [14]. Furthermore, PRV US3 degrades Bclaf1, a key positive regulator of IFN signaling, thereby blocking the binding of ISGF3 to the ISRE. This mechanism is conserved in the HSV‐1 US3 protein [79]. PRV UL24 reduces host antiviral activity by inhibiting the transcription of OASL, a member of the OAS family [53]. Additionally, ISG20 has been shown to inhibit PRV proliferation, and UL24 counteracts this antiviral effect by suppressing ISG20 transcription [80]. Studies have demonstrated that PKR and PKR‐like endoplasmic reticulum kinase (PERK) restrict viral protein synthesis by phosphorylating eukaryotic translation initiation factor 2 alpha (eIF2α). PRV infection attenuates host antiviral responses and enhances viral replication by decreasing PKR expression and inhibiting PERK‐induced eIF2α phosphorylation [81].

In HSV‐1, in addition to the immune evasion mechanisms conserved with PRV described above, other mechanisms exist that regulate the host IFNAR‐JAK‐STAT signaling pathway. Zheng et al. [30] found that UL36USP, a protein encoding a ubiquitin‐specific protease, binds to the receptor IFNAR2, thereby blocking JAK1 binding and inhibiting downstream ISG production, a function independent of the deubiquitinase activity of UL36USP. ICP0 was reported in 2021 to utilize its intrinsic E3 ubiquitin ligase activity to induce K48‐linked ubiquitination and degradation of the host deubiquitinase BRCC36, ultimately leading to the downregulation of IFNAR1 [82]. After the signaling receptor binds to IFN, JAK kinases are activated and phosphorylated. Subsequently, STAT1, phosphorylated by JAK kinases, must translocate to the nucleus and bind to IRF9 to exert its function. As early as 2001, HSV‐1 was reported to inhibit the IFN signaling pathway by suppressing the phosphorylation of STATs and JAKs during the early stage of infection [83]. A study published in 2008 reported that the early protein ICP27 exerts an inhibitory effect on STAT1 phosphorylation and nuclear accumulation. The same study further indicated that IFN‐α‐induced STAT1 phosphorylation levels were moderately reduced in cells infected with an ICP0‐deficient mutant virus, implying that ICP0 also contributes to the partial inhibition of STAT1 phosphorylation [84]. Suppressor of cytokine signaling‐3 (SOCS3) is a host negative regulator of the JAK/STAT pathway, inhibiting JAK phosphorylation by binding to cytokine receptors. Studies have shown that HSV‐1 infection induces high expression of SOCS3 in various cell lines, indirectly inhibiting JAK‐STAT signaling and enhancing viral replication [85], but the specific mechanism of its induction remains unclear.

Studies have shown that VZV infection inhibits the expression of JAK2 and STAT1α, thereby suppressing IFN‐induced ISG expression. In epidermal cells, VZV infection also restricts the nuclear translocation of STAT1/2, and ORF63 plays a crucial role in this process. It not only indirectly prevents ISG induction through transcriptional regulation of viral proteins that interfere with STAT2 phosphorylation but also directly prevents ISG induction by degrading IRF9. Additionally, it can reduce eIF2α phosphorylation, thereby attenuating its restriction on viral protein synthesis [86].

In summary, these three alphaherpesviruses have evolved multiple strategies to inhibit the JAK‐STAT signaling pathway, thereby antagonizing type I IFN‐mediated antiviral immunity (Figure 4).

**Figure 4:**
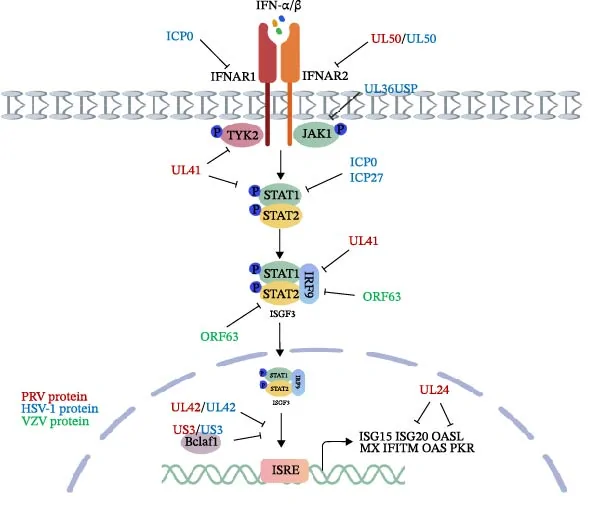
Schematic overview of the IFNAR–JAK–STAT signaling pathway and its inhibition by PRV, HSV‐1, and VZV. IFN‐α/β binding to IFNAR1/IFNAR2 induces receptor dimerization, activating JAK1 and TYK2, which then phosphorylate STAT1 and STAT2. Phosphorylated STAT1/STAT2 form the ISGF3 complex with IRF9, translocate to the nucleus, and bind ISREs to induce ISG transcription. Alphaherpesviruses utilize multiple viral proteins to disrupt interferon (IFN) signaling and the JAK‐STAT pathway at distinct nodes. In PRV, UL50 degrades IFNAR2 via the proteasome pathway (with HSV‐1 UL50 exhibiting conserved function but weaker activity); UL41 (VHS) degrades mRNAs of TYK2, STAT1, and IRF9 through its RNase activity; UL42 binds ISRE via its DNA‐binding domain to block ISGF3 binding (functionally conserved in HSV‐1 UL42); US3 degrades Bclaf1 to block ISGF3‐ISRE interaction (conserved in HSV‐1 US3); and UL24 suppresses OASL and ISG20 transcription. In HSV‐1, additional mechanisms include UL36USP binding to IFNAR2 independent of its deubiquitinase activity to block JAK1 binding; ICP0 utilizes its intrinsic E3 ubiquitin ligase activity to indirectly downregulate IFNAR1, and it also contributes to the inhibition of STAT1 phosphorylation; ICP27 inhibits STAT1 phosphorylation and nuclear accumulation. In VZV, ORF63 inhibits ISG induction by interfering with STAT2 phosphorylation and degrading IRF9.

## 4. Apoptosis

Apoptosis is a form of programmed cell death mediated by caspases and serves as an important host antiviral defense mechanism. The initiation of apoptosis is primarily achieved through two core pathways: the intrinsic pathway, regulated by mitochondrial outer membrane permeabilization (MOMP), which involves pro‐apoptotic members (BIM, PUMA, BID, BMF, NOXA, BIK, BAD, and HRK) and antiapoptotic B‐cell lymphoma 2 (BCL‐2) family members (BCL‐2, MCL‐1, BCL‐XL, BCL‐W, and A1/BFL‐1). Cytochrome C associates with apoptotic protease‐activating factor 1 (APAF‐1) and caspase‐9 to form the apoptosome, which stimulates caspase‐9 activation, which in turn cleaves and thereby activates effector caspases. The second is the extrinsic pathway mediated by death receptors (such as Fas/TNFR1) [87]. This pathway activates pro‐caspase‐8 via TNF receptor type 1‐associated death domain protein (TRADD), receptor‐interacting protein kinase 1 (RIPK1), and Fas‐associated death domain protein (FADD), which then directly or through the mitochondrial pathway activate caspases. Both pathways ultimately converge on the activation of caspases‐3, ‐6, and ‐7, leading to DNA fragmentation, degradation of cytoskeletal proteins, formation of apoptotic bodies, and eventual clearance by phagocytic cells [88]. In the study of alphaherpesviruses, apoptosis is the most comprehensively investigated programmed cell death pathway. On the one hand, viral infection induces apoptosis; on the other hand, viruses regulate apoptosis through various mechanisms to promote their survival, replication, and latency within the host (Figure 5).

**Figure 5:**
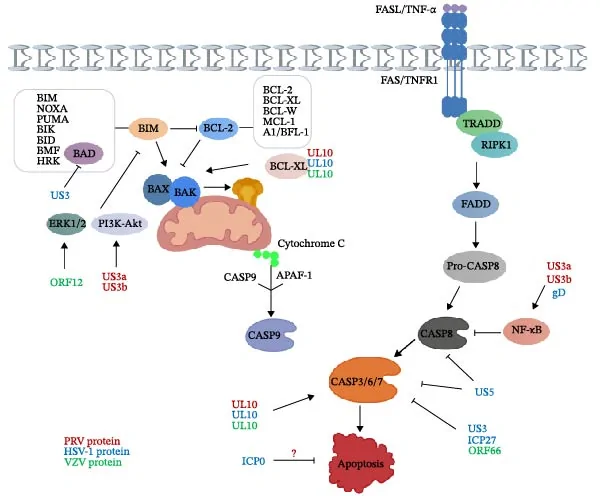
Schematic overview of apoptosis regulation by PRV, HSV‐1, and VZV. During infection, host cells recognize viral nucleic acids and proteins and initiate apoptotic signaling. PRV, HSV‐1, and VZV encode viral proteins that modulate apoptosis at multiple levels to balance host immune defense and viral replication. In PRV, US3a and US3b inhibit apoptosis through the PI3K/Akt and NF‐κB pathways; UL10 induces apoptosis by competitively binding to BCL‐XL to disrupt the BCL‐XL–BAK complex and dissipate mitochondrial membrane potential, and also activates Casp3/7 (both functions are conserved in HSV‐1 and VZV UL10). In HSV‐1, US3 inhibits apoptosis by post‐translationally modifying the pro‐apoptotic protein BAD and blocking caspase‐3 activity; US5 inhibits apoptosis by blocking CASP3 and CASP8 activities; and gD exerts anti‐apoptotic effects through activation of NF‐κB signaling; ICP0 also suppresses apoptosis, though the mechanism remains unknown.. In VZV, ORF66 exerts antiapoptotic effects by inhibiting CASP3; ORF12 inhibits apoptosis by activating ERK1/2, which phosphorylates and inactivates BIM.

### 4.1. PRV and Apoptosis

PRV infection induces and regulates apoptosis in a cell‐type‐dependent manner. Apoptosis can be induced in fibroblasts, epithelial cells, and immune cells. Specifically, in PK15 cells, PRV upregulates pro‐apoptotic Bcl‐2 family members, activates caspase‐3/‐6/‐9, and cleaves poly (ADP‐ribose) polymerase (PARP) [89]. Studies in HEK293T cells have revealed that the PRV glycoprotein gM (encoded by UL10) promotes apoptosis at late stages of infection: gM competitively binds to BCL‐XL, disrupting the BCL‐XL–BAK complex, which leads to BAK oligomerization and BAX activation, dissipation of the mitochondrial membrane potential, and subsequent activation of caspase‐3/7. This mechanism is conserved among gM homologs in HSV‐1, HCMV, EHV‐1, and VZV. Deletion of UL10 in either PRV or HSV‐1 attenuates viral pathogenicity in mice, accompanied by reduced apoptosis and viral replication [90]. Although PRV triggers pro‐apoptotic signals, the virus actively suppresses apoptosis during early infection to facilitate replication [91, 92]. The ATP‐binding site within the kinase domain of US3 is critical for its antiapoptotic function [93]. A 2013 study demonstrated that in ST cells, two US3 isoforms (US3a > US3b) exert antiapoptotic effects in a kinase activity‐dependent manner, localize to mitochondria, activate the Akt and NF‐κB pathways, and confer resistance to apoptosis induced by other viruses [94]. Furthermore, in PK‐15 cells and T cells, gE inhibits apoptosis at the early stages of infection via the ERK1/2‐Bim axis to promote viral replication [92]. In neurons, although PRV infection induces pathological changes, it does not trigger apoptosis [95]. Studies have shown that PRV infection in trigeminal ganglia upregulates antiapoptotic signaling molecules, including Akt, PDK‐1, and IκBα [94].

### 4.2. HSV‐1 and Apoptosis

HSV‐1 also exhibits cell‐type and mechanism specificity in regulating apoptosis. In epithelial cells, the virus inhibits apoptosis at the early stage of infection to gain time for replication while inducing apoptosis at the late stage to facilitate the release of progeny virions [96]. Current studies have identified multiple viral proteins involved in this process, including gM, ICP27, US3, and ICP0. The regulatory function of gM is consistent with that of PRV gM [90]. Regarding ICP27, current studies reveal differential effects across various epithelial cell types. In hTert RPE‐1 cells, ICP27 promotes caspase‐3/7 activation and upregulates caspase‐9 [97]. HEp‐2 cells infected with an ICP27‐null virus (but not the wild‐type virus) undergo apoptosis, characterized by plasma membrane blebbing and formation of apoptotic bodies, in a caspase‐3‐dependent manner [98]. In HeLa cells, ICP27 directly activates p38 signaling to promote host cell apoptosis [99]. In HEK293T cells, ICP27 interacts with 14‐3‐3θ and promotes Bax translocation to the mitochondria by inhibiting the 14‐3‐3θ–Bax interaction, thereby inducing apoptosis [100]. US3 is a well‐characterized antiapoptotic protein. As early as 1999, it was demonstrated that a recombinant virus lacking most of the US3 gene could induce apoptosis in Vero cells, whereas a US3‐repaired virus failed to do so. US3‐deficient viruses retain partial antiapoptotic activity, suggesting that US3 and US5 jointly suppress apoptosis during HSV‐1 infection in Vero cells. Mechanistically, US3 inhibits apoptosis by posttranslationally modifying the pro‐apoptotic protein BAD and blocking caspase‐3 activity. US5 encodes glycoprotein gJ, which blocks apoptosis induced by Fas ligand, ultraviolet irradiation, or granzyme B through inhibition of caspase‐3 and caspase‐8 activities [101]. ICP0 has also been found to induce apoptosis in HEp‐2 cells and Vero cells, although the underlying mechanism remains unknown [102]. Moreover, this function has not yet been verified for PRV ICP0. In immune cells and fibroblasts, HSV‐1 infection induces robust apoptosis, through which the host restricts viral replication [103]. Nevertheless, US3 and US5 exert the same antiapoptotic functions in Jurkat cells as described above [101]. In U937 cells, gD not only binds to HVEM to activate the NF‐κB signaling pathway and upregulate antiapoptotic proteins but also alone inhibits Fas‐mediated apoptosis [104, 105]. In sensory neurons latently infected with HSV‐1, apoptosis is typically absent, allowing the virus to establish a long‐term “coexistence” while awaiting reactivation. The underlying mechanisms include that in SH‐SY5Y neuroblastoma cells, ICP27 reduces caspase‐3/7 activity and restricts caspase‐9 induction [97]. In trigeminal ganglion neurons and human sensory neurons, ICP34.5 is a key neurovirulence factor and plays an essential role in achieving efficient reactivation in an IRF3/7‐dependent manner [54, 106, 107]. An HSV‐1 mutant lacking gD induces apoptosis in human SK‐N‐SH cells, confirming the antiapoptotic function of gD in neuronal cells [108].

### 4.3. VZV and Apoptosis

Apoptosis regulated by VZV also exhibits cell type specificity. In skin cells (such as MeWo cells and human foreskin fibroblasts [HFF]) and immune cells (T cells, B‐cells, and monocytes), VZV infection induces apoptosis [109]. For instance, VZV infection in MeWo cells leads to downregulation of Bcl‐2, cytochrome c release, and cleavage/activation of caspase‐3/8/9 [110]. However, antiapoptotic regulatory strategies also exist. In epithelial cells and fibroblasts, ORF12 triggers ERK1/2 phosphorylation, leading to BIM phosphorylation and subsequent inhibition of apoptosis [111, 112]. An important implication of these findings is that they establish a complete link between the ERK pathway, BIM phosphorylation, and antiapoptotic function. In T cells, ORF66 was found to exert a specific antiapoptotic activity. A virus carrying a premature termination mutation in ORF66 resulted in a tenfold increase in cleaved caspase‐3 levels in T cells, whereas this effect was not observed in melanoma cells or fibroblasts [113]. However, in primary dorsal root ganglion (DRG) neurons, intact fetal ganglia, the severe combined immunodeficiency‐human (SCID‐hu) mouse model, and neurons differentiated from neural stem cells, VZV does not induce apoptosis. Instead, it upregulates antiapoptotic genes such as baculoviral IAP repeat‐containing protein 5 (BIRC5) and proteins including Bcl‐2, Bcl‐XL, and mitochondrially encoded cytochrome c oxidase II (MT‐CO2) while downregulating cleaved caspase‐3, indicating that VZV actively suppresses apoptosis in neurons to facilitate latent infection and reactivation [114]. Additional studies have shown that in SH‐SY5Y cells, a virus lacking ORF63 induces apoptosis, whereas overexpression of ORF63 inhibits both intrinsic and extrinsic apoptotic pathways (e.g., upon NGF withdrawal, staurosporine treatment, or FasL stimulation) [114].

In summary, PRV and HSV‐1 share a common pattern in apoptosis regulation—cell‐type‐dependent inhibition at early stages and induction at late stages—while VZV’s regulation appears more complex and cell‐type‐specific, with evidence of active suppression in neurons but no clear demonstration of late‐stage induction. Table S2 summarizes the research findings on the regulation of apoptosis by three different viruses in various cell types.

## 5. Autophagy

Autophagy serves not only to clear excess intracellular components but also as a host defense mechanism against viral infection. Autophagy consists of three stages: initiation, elongation, and maturation. During initiation, under conditions of energy stress, AMP‐activated protein kinase (AMPK) reverses the inhibition imposed by mechanistic target of rapamycin complex 1 (mTORC1), activating UNC‐51‐like kinase 1 (ULK1) and the PI3K complex, thereby promoting phagophore formation and engulfment of substrates. The elongation stage involves two ubiquitin‐like conjugation systems (autophagy‐related gene ATG7/ATG10 and ATG3/ATG4) that convert microtubule‐associated protein 1 light chain 3 (LC3) into membrane‐bound LC3‐II, driving phagophore expansion. Finally, the closed autophagosome fuses with the lysosome to form an autolysosome, where the contents are degraded by acidic hydrolases [115, 116]. During viral infection, viruses not only induce autophagy but also regulate the interplay between autophagy and host immunity to facilitate their entry, replication, and spread (Figure 6).

**Figure 6:**
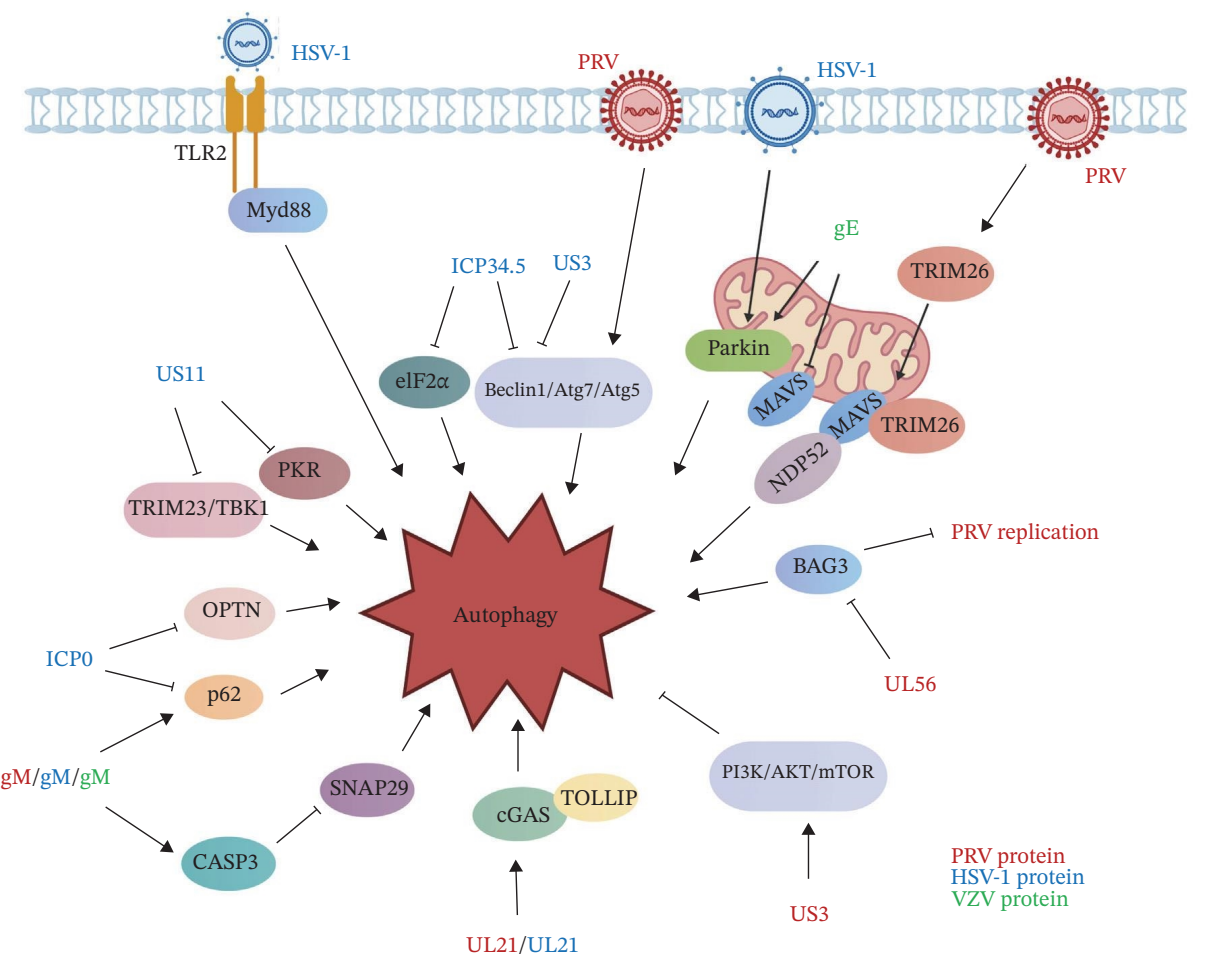
Schematic overview of autophagy regulation by PRV, HSV‐1, and VZV. Autophagy is an intrinsic host defense mechanism against viral infection. PRV, HSV‐1, and VZV infection induce autophagy via distinct pathways, while viral proteins also manipulate the autophagy–immunity crosstalk to facilitate viral entry, replication, and dissemination. During the early stage of infection, PRV induces autophagy through the canonical Beclin‐1‐Atg7‐Atg5 signaling pathway in neuronal cells. PRV infection promotes the association of tripartite motif‐containing protein 26 (TRIM26) with MAVS and its degradation via NDP52‐mediated autophagy and also triggers Parkin‐mediated mitophagy to degrade MAVS. In PRV, US3 inhibits autophagy through the PI3K/AKT/mTOR pathway, while gM induces selective autophagy via SQSTM1/p62 and activates CASP3 to degrade SNAP29, leading to incomplete autophagy (these functions are conserved in HSV‐1 and VZV gM). UL21 degrades cGAS through TOLLIP‐mediated autophagy (functionally conserved in HSV‐1). BAG3 suppresses PRV replication, whereas UL56 interacts with BAG3 and promotes its degradation via the lysosomal pathway. HSV‐1 early infection induces autophagosome formation through the TLR2‐MyD88 signaling pathway and also activates Parkin‐dependent mitophagy. In HSV‐1, ICP34.5 inhibits autophagy through eIF2α and Beclin1; US11 blocks the formation of the TRIM23‐TBK1 complex to suppress autophagy‐mediated antiviral immune responses, and also inhibits autophagosome formation by binding to PKR; ICP0 inhibits the autophagy adaptor proteins p62 and OPTN; US3 directly phosphorylates Beclin1 through its intrinsic kinase activity to inhibit autophagy. In VZV, gE promotes viral replication by facilitating Parkin‐dependent mitophagy.

### 5.1. PRV and Autophagy

During the early stage of infection, PRV induces autophagy through the canonical Beclin‐1‐Atg7‐Atg5 signaling pathway in neuronal cells, a process that is independent of viral replication. Additionally, PRV infection significantly activates the Wnt/β‐catenin signaling pathway, promoting β‐catenin nuclear translocation. This pathway may enhance viral proliferation by upregulating virus‐induced autophagy, whereas inhibition of this pathway reduces PRV titers [117]. However, as infection progresses, multiple PRV‐encoded proteins begin to suppress autophagy levels. Among them, the US3 protein reduces autophagy by activating the PI3K /AKT (protein kinase B)/mTOR (mechanistic target of rapamycin) pathway, which facilitates cell survival and promotes viral replication [118]. The late envelope glycoprotein gM induces SQSTM1/p62 (sequestosome 1)‐dependent selective autophagy while simultaneously activating caspase‐3 (CASP3) to degrade synaptosomal‐associated protein 29 (SNAP29), thereby blocking autophagosome‐lysosome fusion and resulting in incomplete autophagy that protects viral proteins from degradation. This mechanism is highly conserved among gM homologs of other herpesviruses, including HSV‐1, HCMV, EHV‐1, and VZV [119]. On the other hand, PRV also utilizes the autophagic pathway to degrade host antiviral immune molecules. The tegument protein UL21 recruits the E3 ligase UBE3C to catalyze K27‐linked ubiquitination of cGAS, which is subsequently recognized by the cargo receptor TOLLIP and targeted for degradation via the autophagolysosomal pathway, thereby inhibiting the cGAS‐STING‐mediated type I IFN signaling pathway [10]. In addition, TRIM26 facilitates PRV infection by mediating NDP52‐dependent autophagic degradation of MAVS [120], whereas PINK1/Parkin‐mediated mitophagy promotes viral replication by eliminating damaged mitochondria and mitochondrion‐associated MAVS [121], thereby suppressing the IFN response. Although these two autophagic pathways operate through distinct mechanisms, they ultimately converge on MAVS degradation, cooperatively dampening the host innate immunity and enhancing PRV proliferation. Regarding regulatory factors, BAG3 (Bcl‐2‐associated athanogene3), a protein involved in selective autophagy, inhibits PRV replication, whereas the PRV UL56 protein interacts with BAG3 and promotes its degradation via the lysosomal pathway. However, this phenomenon varies among different PRV strains, suggesting the involvement of other viral proteins in the regulation [122].

### 5.2. HSV and Autophagy

During the early stage of infection, HSV‐1 induces autophagy to promote its own replication. Studies have shown that in THP‐1 monocytes and primary human monocytes, early HSV‐1 infection induces autophagosome formation through the MyD88/TLR2 signaling pathway, independent of viral gene expression. Inhibition of autophagy reduces viral yield, suggesting that HSV‐1 utilizes the autophagic machinery to promote its replication in these semipermissive cells [123]. Furthermore, early HSV‐1 infection activates Parkin‐dependent mitophagy, which is inhibited at the late stage of infection, indicating a complex interplay between HSV‐1 infection and autophagy [124].

ICP34.5 [107] is a well‐characterized multifunctional autophagy inhibitory factor in HSV‐1. By directly binding to Beclin1, it inhibits Beclin1‐dependent autophagy, a function crucial for the maintenance of neurovirulence. ICP34.5 can also bind to protein phosphatase 1 alpha (PP1α) to dephosphorylate eIF2α, preventing host translational shutoff and indirectly inhibiting autophagy. Additionally, it alters mitochondrial localization by affecting mitochondrial transport (e.g., through interaction with phosphoglycerate mutase family member 5 [PGAM5]), thereby regulating the balance between autophagy and apoptosis [125]. The US11 protein binds to the ADP‐ribosylation factor (ARF) domain of tripartite motif‐containing protein 23 (TRIM23), blocking the formation of the TRIM23‐TBK1 complex and inhibiting autophagy‐mediated antiviral defense [126]. US11 can also bind to PKR, suppressing autophagosome formation induced by poly(I:C) or starvation [127]. Furthermore, it promotes the accumulation of the active caspase‐8 fragment p18, which subsequently cleaves autophagy‐related protein 3 (Atg3), thereby blocking autophagy [128]. ICP0 downregulates two autophagic adaptor proteins, p62 and optineurin (OPTN), in a proteasome‐dependent manner; however, its E3 ubiquitin ligase activity is not required for this function, and the precise mechanism requires further investigation [129]. US3, through its intrinsic kinase activity, directly phosphorylates Beclin1, inhibiting autophagy and thereby promoting viral replication in nonneuronal cells [130]. Additionally, studies have suggested that ICP22 may indirectly affect autophagy through interaction with P53 [131].

### 5.3. VZV and Autophagy

In fibroblasts (e.g., MRC‐5) [132], keratinocytes, and human skin xenograft models, VZV infection upregulates autophagy, as evidenced by increased autophagosome formation and the conversion of LC3‐I to LC3‐II (microtubule‐associated protein 1 light chain 3‐I to ‐II). This upregulation begins at 48 h postinfection and persists even when late viral gene expression is blocked, suggesting that autophagy induction is primarily driven by cellular stress responses to viral entry rather than specific activation by a single viral protein. In neuronal cells (human SH‐SY5Y neurons), VZV infection also induces autophagy (manifested by increased LC3‐II conversion and colocalization of viral glycoprotein E with p62/SQSTM1), but here, autophagy exerts an antiviral effect: autophagy inducers reduce VZV transcription levels, whereas autophagy inhibitors enhance viral gene expression [133]. Recent studies have revealed that VZV actively induces PINK1/Parkin‐dependent mitophagy to promote its own replication. Viral glycoprotein E (gE) plays a critical role in this process: gE interacts with LC3 and upregulates mitochondrial reactive oxygen species (ROS), thereby promoting PINK1/Parkin‐mediated mitophagy. This process also inhibits MAVS oligomerization and STING translocation, thereby blocking MAVS‐ and STING‐mediated IFN production. In a human skin organ culture model, induction of mitophagy similarly enhances VZV replication and attenuates the IFN response. Thus, VZV gE achieves evasion of the innate immune pathway by activating PINK1/Parkin‐dependent mitophagy [35]. In 2015, Buckingham et al. [134] examined VZV‐induced autophagic flux using two approaches: radiolabeled protein analysis and dual‐color LC3 plasmid assay and found no evidence of autophagic blockage. This study also demonstrated that VZV‐induced autophagy, compared to HSV‐induced autophagy, lacks two modulatory factors, ICP34.5 and US11 [134]. In a 2018 study, researchers constructed a recombinant VZV carrying the HSV‐1 ICP34.5 gene. They found that infection with this recombinant virus did not significantly alter the autophagic response in the skin compared to infection with wild‐type VZV [135]. Together with the 2015 report, these findings indicate that ICP34.5 is a unique immune evasion and neurovirulence factor of HSV‐1 rather than a common feature of alphaherpesviruses. To date, no VZV‐encoded autophagy‐inhibitory gene has been identified.

Based on the available findings, all three herpesviruses exhibit complex interactions with the host autophagic pathway and utilize or manipulate autophagy to promote their own replication and immune evasion. For example, all three viruses induce PINK1/Parkin‐mediated mitophagy; both PRV and HSV encode multiple autophagy‐inhibitory proteins; and all three viruses activate caspase‐3 via the gM protein to degrade SNAP29, thereby blocking autophagosome‐lysosome fusion and resulting in incomplete autophagy. However, overall, the current understanding of the mechanisms by which these viruses regulate autophagy remains insufficient, particularly regarding the viral proteins involved in autophagy regulation in VZV, which have been poorly studied.

## 6. Imbalance in Research Status: Disparities in Immune Evasion Studies Among PRV, HSV‐1, and VZV

Based on the foregoing comparative analysis, the three alphaherpesviruses exhibit markedly different levels of research maturity. This section systematically delineates the known, underexplored, and entirely uncharacterized domains for each virus with the aim of informing future investigative directions.

### 6.1. Comparative Depth of Research on Individual Viruses

#### 6.1.1. PRV: A Model Virus With Extensive Research Coverage

In the cGAS‐STING pathway, 11 viral proteins have been identified as regulators (UL42 [14], US1 [15], UL21 [10], UL49 [16], ICP0 [18], UL13 [19], UL38 [20], US2 [21], UL48 [22], UL24 [23], and UL41 (VHS) [24]), with the majority characterized to the level of functional domains or enzymatic activities. In the JAK‐STAT pathway, more than five viral proteins have been implicated in modulation. Despite this depth, notable gaps persist: no PRV‐encoded protein has been demonstrated to directly target receptors or adaptors in the TLR or RLR signaling cascades; the specific E3 ubiquitin ligase responsible for UL24‐mediated p65 degradation remains unidentified [65]; and the mechanistic basis for ICP0’s antiapoptotic function is entirely unknown [18].

#### 6.1.2. HSV‐1: Mechanistic Diversity

HSV‐1 not only has the largest number of identified proteins in each individual pathway, but also exhibits mechanistic diversity in its regulation of various pathways. Unique strategies include UL56‐mediated suppression of the cGAMP channel [25], UL37‐mediated deamidation of cGAS [26], UL36USP‐mediated deubiquitination of STING and direct binding to IFNAR2 [30], ICP27‐mediated blockade of STAT1 phosphorylation and nuclear import [84], ICP0‐mediated indirect downregulation of IFNAR1 via BRCC36 [82], ICP0‐mediated p50 degradation [64], US11‐mediated disruption of the TRIM23‐TBK1 complex [126], and ICP34.5‐mediated inhibition of autophagy [125]. However, several aspects remain unresolved: the precise mechanism by which UL49 suppresses cGAS has not been fully elucidated; the direct substrate of US3 in the TLR2 pathway awaits identification; and whether HSV‐1 employs mitophagy to degrade MAVS has not been systematically examined.

#### 6.1.3. VZV: Mechanistic Understanding Remains Limited

In the cGAS‐STING pathway, only four proteins have been functionally characterized, leaving over 90% of the 71 ORFs unexplored in this critical antiviral axis. In the TLR pathways, no VZV‐encoded protein has been definitively demonstrated to modulate signaling. For the JAK‐STAT pathway, only ORF63 has been reported to exert regulatory activity [86], and no VZV protein has been identified that targets JAK kinases or the IFNAR receptor. Most notably, VZV lacks identified autophagy inhibitors (with only two identified)—a striking contrast to PRV and HSV‐1—given that autophagy serves as a protective antiviral mechanism in neurons where VZV establishes latent infection. Table 1 summarizes and compares the research richness of the three viruses across various pathways.

**Table 1:** Summary and Comparison of research depth on immune evasion pathways among PRV, HSV‐1, and VZV.

Category	PRV	HSV‐1	VZV
Identified proteins in cGAS‐STING pathway	11	14	4
Defined proteins in TLR pathway	None	2	None
Identified proteins in RLR pathway	2	5	1
Identified proteins in NF‐κB pathway	4	7	2
Identified proteins in JAK‐STAT pathway	5	6	1
Number of anti‐apoptotic proteins	2	6	3
Number of autophagy‐inhibitory factors	4	6	2
In vivo validation in natural host	Yes (pig)	Limited	None

The stark asymmetry in research depth across the three viruses, as quantified above, is not merely a reflection of scientific interest but is largely attributable to the inherent biological differences. Specifically, VZV’s strict human tropism and the consequent lack of robust small‐animal models have severely constrained mechanistic studies, forcing researchers to rely on less physiologically relevant cell culture systems. In contrast, the broad host range of PRV and the availability of well‐established murine and primate models for HSV‐1 have greatly facilitated their in‐depth characterization. This fundamental disparity in experimental tractability must be borne in mind when interpreting the current state of knowledge.

### 6.2. Shared Critical Unresolved Questions

Conservation of mitophagy‐mediated MAVS clearance: this mechanism has been demonstrated in PRV and VZV, but whether HSV‐1 employs the PINK1/Parkin pathway to eliminate MAVS remains unknown; structural basis for functional differences among homologous proteins: although both PRV UL50 and HSV‐1 UL50 degrade IFNAR2 [76], they exhibit markedly different efficiencies, yet the structural determinants underlying this disparity are unidentified. Similarly, the mechanistic basis for the functional divergence between PRV ICP0 and HSV‐1 ICP0 in NF‐κB regulation awaits elucidation [63, 64]; coordinated regulation of apoptosis and autophagy: all three viruses can link these two pathways through gM protein [90, 119], but how they dynamically coordinate these processes across different cell types and infection stages remains poorly understood; immune evasion in neurons: although neurons serve as the primary site of latency for all three viruses [136], the vast majority of mechanistic data have been derived from fibroblasts or epithelial cell lines, leaving the neuronal context relatively underexplored: whether VZV encodes functional analogs of key PRV/HSV‐1 immune evasion factors: it remains entirely unknown whether VZV encodes a ribonuclease analogous to PRV UL41/VHS [24, 78], an autophagy inhibitor analogous to HSV‐1 ICP34.5 [125], or a protein capable of degrading JAK kinases.

## 7. Limitations of Current Methodological Approaches and Their Impact on Research Findings

Beyond the differential analysis of research outcomes for each virus, it is equally important to critically examine the experimental methodologies upon which current mechanistic studies are predicated. The vast majority of findings summarized in this review derive from in vitro cell culture systems, and this methodological homogeneity substantially compromises the generalizability and physiological relevance of the conclusions drawn.

### 7.1. Predominance of In Vitro Cell Models and Their Inherent Limitations

The preponderance of mechanistic discoveries has been generated using immortalized or transformed cell lines—such as HEK293T, Vero, PK15, and HeLa cells—which are selected for their high transfection efficiency, permissiveness to viral infection, and experimental tractability rather than for their physiological relevance to the natural target tissues of these viruses. These cell lines differ fundamentally from the primary targets of alphaherpesvirus infection in several critical aspects: (i) they lack the three‐dimensional architecture and cell–cell interactions characteristic of neuronal or epithelial tissues; (ii) they exhibit altered expression profiles of innate immune sensors and signaling adaptors; and (iii) they fail to recapitulate the metabolic and stress conditions encountered in vivo.

The ramifications of this limitation are evident in several contexts. For PRV, key findings concerning UL50 [76] and UL21 [10] were predominantly obtained in HEK293T or PK15 cells, yet whether these mechanisms operate in neural tissues of the natural porcine host remains unverified. For VZV, the absence of suitable small‐animal models has compelled researchers to rely on human fibroblasts [132] or melanoma cells [109] (e.g., MRC‐5, HFF, and MeWo), neither of which adequately recapitulates the neuronal or cutaneous microenvironment where VZV exerts its pathogenic effects. The fact that only four VZV proteins have been identified in the cGAS‐STING pathway may reflect not only a genuine paucity of such mechanisms but also the technical constraints imposed by studying VZV in physiologically relevant cell types. For HSV‐1, although murine models and primary neuronal cultures are more readily available than for VZV, the vast majority of mechanistic insights—particularly those concerning immediate‐early protein functions—originate from nonneuronal cell lines, raising legitimate concerns about their applicability to latently infected sensory neurons.

### 7.2. Host Species Specificity and Cross‐Species Generalizability

A related yet distinct issue concerns host species specificity. PRV serves as a paradigmatic example: although its broad host range is frequently cited as an advantage for its use as a model virus, this very property complicates the interpretation of mechanistic data. Viral proteins identified as immune evasion factors in murine macrophage cell lines may operate through pathways that are not conserved in porcine or human cells owing to substantial species‐specific differences in certain innate immune signaling components. Conversely, mechanisms discovered in porcine kidney cells may not extrapolate to the clinically relevant cell types in PRV pathogenesis—namely, porcine neurons. To date, systematic validation of PRV immune evasion mechanisms in porcine neural tissues or human cells remains scarce. For HSV‐1 and VZV, both of which are human‐specific pathogens in their natural hosts, the use of primate or human cell lines is conceptually more appropriate. However, the cell types employed (fibroblasts and epithelial cells) frequently deviate from the primary targets (neurons and keratinocytes), and the experimental context (in vitro acute infection) does not recapitulate the physiological conditions of latency and reactivation.

### 7.3. Depth of Mechanistic Characterization: From Phenotype to Molecular Resolution

Another methodological disparity manifests in the varying depths of mechanistic characterization across different viral proteins. Certain proteins—notably PRV UL21 [10], UL42 [14], and HSV‐1 ICP34.5 [125]—have been investigated to the level of specific enzymatic activities, interaction interfaces, or ubiquitin linkage types, whereas many others remain characterized only at the phenotypic level. For instance, PRV ICP0 (EP0) has been reported to degrade cGAS through an AKT‐mTOR‐dependent autophagolysosomal pathway [18], yet its direct molecular interactors and the precise E3 ligase involved have not been identified. Similarly, while the antiapoptotic activity of HSV‐1 ICP0 is known to exist [102], the underlying mechanism remains unelucidated. This unevenness in characterization depth across the viral proteome impedes the comprehensive construction of mechanistic maps.

### 7.4. Methodological Recommendations

In light of the aforementioned limitations, future investigations should prioritize the following methodological refinements: (i) validation of key immune evasion mechanisms in primary cells derived from the natural host species and target tissues (e.g., porcine trigeminal ganglion neurons for PRV and human keratinocytes and sensory neurons for HSV‐1 and VZV); (ii) confirmation, where feasible, in ex vivo tissue explants or in vivo infection models, with particular attention to the latent and reactivation phases; (iii) systematic side‐by‐side comparisons of homologous proteins from different viruses under identical experimental conditions and cell types, to eliminate confounding variables arising from assay system discrepancies; and (iv) incorporation of structural biology approaches (e.g., cryo‐electron microscopy, X‐ray crystallography, or AlphaFold‐based predictions) to advance beyond phenotypic observations and establish the physical basis of protein–protein interactions. Addressing these methodological gaps is essential for translating mechanistic insights into therapeutically actionable targets.

## 8. Conclusions and Future Perspective

In summary, research on the innate immune evasion strategies of PRV, HSV‐1, and VZV has progressed beyond phenomenological description and entered an era of in‐depth mechanistic dissection. The foregoing comparative analysis reveals both notable divergences and commonalities in their immunomodulatory strategies. In summary, research on the innate immune evasion strategies of PRV, HSV‐1, and VZV has progressed beyond phenomenological description and entered an era of in‐depth mechanistic dissection. The foregoing comparative analysis reveals both notable divergences and commonalities in their immunomodulatory strategies. Based on the available findings, PRV and HSV‐1 exhibit the most systematic multinode, multilayered regulatory networks across individual pathways, particularly in the cGAS‐STING and JAK‐STAT axes. In addition, HSV‐1 possesses the most extensive repertoire of protein tools, with relatively prominent research in the realms of apoptosis modulation and autophagy inhibition. Research on VZV, however, lags considerably behind: aside from a few proteins such as ORF9 [34], ORF63 [86, 114, 137], ORF61 [72], ORF66 [113, 138], ORF12 [111], and gE [35], the vast majority of its ORFs remain entirely uncharacterized with respect to immune regulation, and in the TLR, not a single VZV‐encoded protein has been definitively identified. Building upon these comparative findings, future investigations may be advanced along four priority directions, which form a progressive continuum from fundamental mechanistic elucidation to translational exploration.

### 8.1. First Priority

Systematic Filling of the Knowledge Gaps in VZV Immune Evasion Mechanisms. Priority questions include (i) the precise mechanism by which ORF63 modulates STAT2 phosphorylation and IRF9 degradation in neurons and its relevance to latent infection; (ii) the detailed steps by which gE mediates PINK1/Parkin‐dependent mitophagy to degrade MAVS and the role of this pathway in cutaneous primary infection; and (iii) whether additional VZV ORFs participate in MAVS clearance and whether this mechanism is conserved in HSV‐1. Physiologically relevant models, such as human neurons and skin organoids, are recommended for these investigations.

### 8.2. Second Priority

Systematic cross‐functional validation of homologous viral proteins. Homologous proteins across different viruses exhibit both conservation and functional divergence. For instance, PRV UL50 degrades IFNAR2 more potently than its HSV‐1 ortholog [76], yet the structural basis for this difference remains unknown. Moreover, PRV ICP0 (EP0) directly binds p65 [63], whereas HSV‐1 ICP0 degrades p50 [64]—fundamentally divergent strategies in NF‐κB regulation. Key homologous proteins—including UL21, US3, UL50, and gM—should be compared in parallel within identical experimental systems. These analyses should be integrated with structural approaches such as AlphaFold2‐based predictions, crystallography, or cryo‐electron microscopy to resolve complex structures, complemented by point mutagenesis to validate functional domains, thereby elucidating the molecular underpinnings of functional diversification.

### 8.3. Third Priority

In‐depth elucidation of the dynamic regulatory mechanisms by which viruses coordinate autophagy and apoptosis. All three viruses, through gM, activate the mitochondrial apoptosis pathway at the late stage of infection by competitively binding to BCL‐XL; meanwhile, they block autophagic flux by activating CASP3 to cleave SNAP29, leading to incomplete autophagy [90, 119]. Moreover, PRV and VZV have been demonstrated to exploit mitophagy for MAVS clearance [35, 121] (HSV‐1 remains to be verified). However, how viruses dynamically modulate these pathways in a context‐dependent manner—switching between neurons and epithelial cells, or between acute and latent infection—remains largely unexplored. Future efforts should: (i) verify the conservation of mitophagy‐mediated MAVS clearance in HSV‐1 and identify the viral proteins responsible; (ii) employ dual‐fluorescence live‐cell reporter systems to track the spatiotemporal dynamics of apoptosis and autophagy across different cell types and infection stages; and (iii) explore the potential correlation between gM’s dual regulatory functions in apoptosis and autophagy, e.g., whether CASP3‐mediated SNAP29 cleavage indirectly impacts BCL‐XL/BAK complex stability.

### 8.4. Fourth Priority

Exploration of antiviral strategies targeting host immune pathways, with enhanced in vivo and ex vivo validation. Given that more than 10 viral proteins converge on a single pathway (e.g., the 11 PRV proteins in the cGAS‐STING axis), targeting individual viral proteins is prone to engender drug resistance. Therefore, small‐molecule compounds that restore cGAS‐STING or JAK‐STAT function (e.g., STING agonists and rapamycin analogs) should be screened, with careful evaluation of neuronal cytotoxicity and blood–brain barrier permeability. PROTAC technology may be leveraged to design degraders targeting key immune evasion proteins—such as PRV UL41 (VHS) and HSV‐1 ICP34.5—that harness the host ubiquitin‐proteasome system. Most critically, these findings should be validated in ex vivo tissue explants (e.g., porcine trigeminal ganglia for PRV and human skin for VZV) and in vivo infection models, with particular emphasis on the latent and reactivation phases as critical windows for therapeutic intervention. In vivo data are essential to compensate for the current overreliance on cell‐based systems and to provide a pathophysiologically relevant foundation for clinical translation.

In conclusion, future research should be structured around the above four priority directions, forming a progressive investigative continuum: from filling the VZV knowledge gap (foundational), through homologous protein comparisons (mechanistic deepening), to the cross‐regulation of autophagy and apoptosis (system integration), and finally to host‐directed therapy with in vivo/ex vivo validation (translational application). Mechanistic discoveries at each stage should be cross‐validated in physiologically relevant models, thereby transforming mechanistic insights into therapeutically actionable targets. This comprehensive approach will not only systematically address the knowledge asymmetries revealed by the comparative analysis but also provide a robust theoretical framework for understanding alphaherpesvirus pathogenesis and developing novel antiviral strategies.

## Author Contributions

**Aiping Wang**: writing – review and editing, conceptualization, supervision. **Enping Liu**: funding acquisition, methodology, writing – original draft, investigation, software. **Jiayi Xue and Mengting He**: software, methodology. **Yumei Chen and Chao Liang**: investigation, funding acquisition. **Jingming Zhou**: methodology.

## Funding

This research was funded by the Major Projects of the National Nature Science Foundation of China (Grant 32192454), the General Program of the Natural Science Foundation of Henan Province (Grant 252300421401), and the Major Research Program of Longhu Laboratory of Advanced Immunology (Grant LHLab_ZD20230012).

## Disclosure

All authors have read and agreed to the published version of the manuscript. After using BioGDP and DeepSeek, the authors reviewed and edited the content as needed and take full responsibility for the final content of the publication.

## Conflicts of Interest

The authors declare no conflicts of interest.

## Supporting Information

Additional supporting information can be found online in the Supporting Information section.

## Supporting information


**Supporting Information** This section provides supporting data that support the findings of the main study. Table S1: A comparative analysis of the genomic features and protein sequence homology among the three viruses investigated. Table S2: The findings from previous studies on the regulation of apoptosis by these viruses across different cell types, offering a broader context for our results.

## Data Availability

Data sharing is not applicable to this article, as no new data were created or analyzed in this study.
