# Supplementary material for: Divergent Strategies in Innate Immune Evasion: A Comparative Review of Three Alphaherpesvirus Subfamily Members—PRV, HSV‐1, and VZV
Source: Transbound Emerg Dis. 2026 Oct 8;2026:6236477. doi: 10.1155/tbed/6236477 (PMC13647301; doi:10.1155/tbed/6236477)
Supplement: Supplementary file 1 [file TBED-2026-6236477-s001.docx]

Supplementary Table 1. Comparison of genomic information and protein sequence homology among three viruses.

| Virus name |  | PRV | HSV-1 | VZV |
| --- | --- | --- | --- | --- |
| Genomic information | Genome size | 154kb | 152kb | 125kb |
|  | Gene number | ＞70 | ＞80 | ＞71 |
| Homology analysis of key proteins (compared with PRV) | US3 |  | 40% | 38% |
|  | US1 /ICP22 |  | 31% | 50% |
|  | UL21 |  | 31% | 34% |
|  | UL50 |  | 41% |  |
|  | UL42 |  | 30% |  |
|  | ICP0 |  | 37% |  |
|  | UL10/gM |  | 32% | 34% |
|  | UL19 |  | 59% |  |
|  | UL27 |  | 54% |  |
|  | UL49/VP22 |  | 48% |  |
|  | UL13 |  | 30% |  |

* Sequence homology was analyzed using BLASTp. This table only includes homologous proteins that are mentioned in the text.

Supplementary Table 2. Summary of research findings on apoptosis regulation by three viruses in different cell types.

| Cell type | PRV | HSV-1 | VZV |
| --- | --- | --- | --- |
| Fibroblast | In ST cells, US3a/b exerts anti-apoptotic effects through the PDK-1/PI3-K/Akt and NF-κB pathways. | In MEF cells, apoptosis is induced. | In HFF cells and MeWo cells, ORF12 protein triggers ERK1/2 phosphorylation, leading to BIM phosphorylation and thereby inhibiting apoptosis. |
| Epithelial cell | ① In PK-15 cells, PRV infection upregulates pro-apoptotic Bcl-2 family members, activates caspase-3/-6/-9, and cleaves poly(ADP-ribose) polymerase (PARP).  ② In PK-15 cells, gE triggers ERK1/2 phosphorylation, leading to BIM phosphorylation, which inhibits apoptosis at the early stage of infection to promote viral replication.  ③ In HEK293T cells, gM competitively binds to BCL-XL, disrupting the BCL-XL–BAK complex, which results in BAK oligomerization and BAX activation, dissipation of the mitochondrial membrane potential, and subsequent activation of caspase-3/7 to trigger apoptosis. | ① In HEK293T cells, gM competitively binds to BCL-XL, disrupting the BCL-XL–BAK complex, which leads to BAK oligomerization and BAX activation, dissipation of the mitochondrial membrane potential, and activation of caspase-3/7 to trigger apoptosis.  ② In hTert RPE-1 cells, ICP27 promotes caspase-3/7 activation and upregulates caspase-9, thereby facilitating host cell apoptosis.  ③ In HeLa cells, ICP27 directly activates p38 signaling to promote host cell apoptosis.  ④ In HEK293T cells, ICP27 interacts with 14-3-3θ and promotes Bax translocation to the mitochondria by inhibiting the 14-3-3θ–Bax interaction, thereby inducing apoptosis.  ⑤ In Hep-2 and Vero cells, ICP0 exerts an anti-apoptotic effect, although the underlying mechanism remains unknown.  ⑥ In Hep-2 and Vero cells, US3 phosphorylates BAD, thereby blocking caspase-3.  ⑦ In Vero cells, US5 blocks apoptosis by inhibiting caspase-3 and caspase-8 activities. | ① In HEK293T cells, gM competitively binds to BCL-XL, disrupting the BCL-XL–BAK complex, which leads to BAK oligomerization and BAX activation, dissipation of the mitochondrial membrane potential, and activation of caspase-3/7 to trigger apoptosis.  ② In HaCaT cells, ORF63 alone protects cells from STS- and FasL-induced intrinsic and extrinsic apoptosis.  ③ In HEK293T cells, ORF12 triggers ERK1/2 phosphorylation, leading to BIM phosphorylation, thereby inhibiting apoptosis. |
| Neuron | It does not induce apoptosis.  Instead, it upregulates anti-apoptotic signaling molecules (Akt, PDK-1, IκBα) in trigeminal ganglion cells. | It does not induce apoptosis.  ① In SH-SY5Y neuroblastoma cells, ICP27 reduces caspase-3/7 activity and restricts caspase-9 induction.  ② In trigeminal ganglion neurons and human sensory neurons, ICP34.5 is a key neurovirulence factor and is essential for efficient reactivation in an IRF3/7-dependent manner. | It does not induce apoptosis.  ① No apoptosis was detected in primary human DRG neurons, intact fetal ganglia, the SCID-hu mouse model, neurons differentiated from neural stem cells, or iCell neurons.  ② In SH-SY5Y cells, ORF63 inhibits apoptosis induced by both intrinsic and extrinsic pathways. |
| Immune cell | In T cells, gE triggers ERK1/2 phosphorylation, leading to BIM phosphorylation, which inhibits apoptosis at the early stage of infection to promote viral replication. | ① In U937 cells, gD not only binds to HVEM to activate the NF-κB signaling pathway and upregulate anti-apoptotic proteins, but also alone inhibits Fas-mediated apoptosis.  ② In Jurkat cells, US3 inhibits apoptosis by post-translationally modifying the pro-apoptotic protein BAD and blocking caspase-3 activity, while US5 inhibits apoptosis by suppressing caspase-3/8 activities. | In T cells, the ORF66 mutant virus induces a ten‑fold increase in cleaved caspase‑3 levels, yet exerts anti‑apoptotic activity. |
